# Extraction of tungsten from scheelite using hydrodynamic and acoustic cavitation

**DOI:** 10.1016/j.ultsonch.2020.105408

**Published:** 2020-12-07

**Authors:** Örjan Johansson, Taraka Pamidi, Vijay Shankar

**Affiliations:** Engineering Acoustics, Dept of Civil, Environmental and Natural Resources of Engineering, Luleå University of Technology, 971 87 Luleå, Sweden

**Keywords:** Ultrasonic reactor, Acoustic and hydrodynamic cavitation, Tungsten, Scheelite

## Abstract

The primary purpose of this study is to investigate the effects of hydrodynamic and acoustic cavitation (HAC) on the leaching efficiency of tungsten. The aim is to reduce energy use and to improve the recovery rate. The goal is also to carry out a leaching process at a much lower temperature than in an autoclave process that is currently used in the industry. Energy-efficient initiation and collapse of cavitation bubbles require optimization of (i) vibro-acoustic response of the reactor structure, (ii) multiple excitation frequencies adapted to the optimized reactor geometry, and (iii) hydrodynamic cavitation with respect to orifice geometry and flow conditions. The objective is to modify and apply a previously in house developed high power cavitation reactor in order to recover tungsten by leaching of the dissolution of scheelite in sodium hydroxide. In this process, various experimental conditions like dual-frequency excitation, different orifice geometry have been investigated. The numerically optimized reactor concept was excited by two frequencies 23 kHz and 39*–*43 kHz in various flow conditions. The effects of leaching time, leaching temperature, ultrasonic power and geometry of orifice plates have been studied. The leaching temperature was varied from 40 °C to 80 °C. The concentration of leaching reagent sodium hydroxide (NaOH) was 10 mol/L.The results were compared to conventional chemical leaching. Energy supplement with acoustic cavitation of 130 kWh/kg concentrate resulted in a leaching recovery of tungsten (WO_3_) of 71.5%, compared to 36.7% obtained in absence of ultrasound. The results confirm that the method developed is energy efficient and gives a recovery rate potentially better than current autoclave technology.

## Highlights

• Energy efficient and intensified leaching by hydrodynamic and acoustic cavitation.
• The acoustic cavitation intensity is optimized by multiphysics simulation.
• Acoustic cavitation combined with a weak hydrodynamic effect gives two times better results.
• Hydrodynamic and acoustic cavitation improved recovery rate of tungsten by 50%

## 1 Introduction

Tungsten is commonly used in the manufacture of machinery, pharmaceutical, defense industry, etc. Tungsten exists mainly in the form of wolframite and scheelite in nature [1]. Roughly two-thirds of the world’s tungsten reserves contain scheelite deposits [2]. Tungsten from scheelite is currently extracted by the method of decomposing scheelite with sodium hydroxide or carbonate solutions at higher temperatures and pressures, and producing sodium tungstate [3], [4], [5]. These methods have the drawbacks of high temperatures and high pressures and require a relatively high amount of reagent with associated high energy use and increased reagent costs [1], [6].

Under nearly ambient conditions, in order to generate localized high temperatures and pressures hotspots, the cavitation process can be used as a source of energy input in the field of chemical processing [15], [16]. The research work described in this paper aims to enhance the leaching process by combining hydrodynamic and acoustic cavitation to reduce energy use and recovery time and to increase the recovery rate. Ultrasound controlled cavitation is known to generate an accelerated leaching process with a higher yield at a lower temperature compared to existing technologies [7]. In the past several decades, ultrasound has become more common in the leaching of hydrometallurgy [8], [9]. Ultrasound has been proven to be useful in improving the leaching effectiveness and product yields [10], [11], [12]. The challenges are linked to up-scaling, robustness, and energy efficiency. The investigation focuses on scheelite concentrate (CaWO_4_), which requires extreme leaching conditions with respect to temperature and pressure. The process is therefore energy-intensive.

The objective is to optimize the process with a previously in-house developed ultrasonic reactor designed for high cavitation intensity by adding a hydrodynamic device (orifice plates) and varying excitation frequencies, flow conditions, temperature and input power. The project goals are to:

1. Optimize recovery and kinetics of leaching according to temperature, pressure, input electrical power, excitation frequency, solid concentration, and particle size.
2. Modify the reactor design principle based on multi-physical simulation and sensitivity analysis with respect to the leaching reagent and mineral particles.

### 1.1 Leaching by hydrodynamic and acoustic cavitation

Intensification of leaching by high power ultrasound in order to generate transient cavitation has a strong potential for various applications in mineral processing and hydrometallurgy. Metal extraction by a leaching process can be more efficient with ultrasound assistance. High intensity acoustic cavitation creates very high local temperatures, which increases the solubility and diffusivity, and high pressures, which favor penetration and transport when cavitation bubbles form and collapse close to particle surfaces. When cavitation occurs, the cavity collapse near the particle is asymmetrical, and high-speed liquid jets are generated during the process. However, parallel to the production of micro-jet effects clouds of bubbles collapse, generating strong shockwaves in the fluid. The effect on the particle surface of these jets and shock waves is very strong and can after interaction produce newly exposed and highly reactive surfaces. Based on these properties ultrasound has the potential to be beneficial in hydrometallurgy by improving both leaching kinetics and recoveries. The following two cases can be identified where ultrasound could improve leaching:

### 1.2 Case 1

Leaching of minerals where leaching proceeds through the surface reaction controlled mechanism characterized by high activation energy [28], [29]. Minerals that fall into this category are tetrahedrite, nickel laterites, scheelite, wolframite, etc. These types of minerals are typically leached in autoclaves at elevated pressures (up to 20 bars) and temperatures (up to 240 °C) making them highly energy-intensive. Other examples are metallurgical by-products like slag, dust, etc., where speiss formed in base metal production is one example. Hence, leaching at lower temperatures and pressures leads to cost effective and energy-saving opportunities [7].

### 1.3 Case 2

Leaching processes where leaching kinetics are slow because of the formation of diffusion layers on the surface of the particles to be leached, i.e. through a diffusion controlled mechanism. This is the case for leaching of chalcopyrite in sulfate solution where the chalcopyrite surface with time is known to be passivated by the formation of a surface layer resulting in low copper recoveries. Another example is during cyanide leaching for gold extraction, where the gold particles sometimes get covered by clay or oxide layers. In these cases, ultrasound can remove these layers and give higher metal recoveries during leaching [13].

Despite these generally accepted benefits of ultrasound, assisted leaching the method is not practiced in hydrometallurgical processes. This is because the controlled cavitation is not fully developed and problems are encountered in the implementation on a larger scale. One limitation often seen, relates to the use of sonotrodes or horns directly inserted in the fluid. Typically, when the power delivered to the reaction mixture increases, the rate of the reaction increases to a maximum and then decreases with a continued increase in power [5]. A possible explanation for the observed decrease at high power input is the formation of a dense cloud of cavitation bubbles near the probe tip that acts to block the energy transmitted from the probe to the fluid [1], [6]. Another problem is that the leaching reagent and multi-phase flow conditions may alter the interconnected acoustic, mechanical and electrical impedances of the reactor system, which changes the resonance frequency and thereby input power [30].

Achieving transient cavitation requires an understanding of how different excitation mechanisms and resonance concepts can interact and be optimized [15], [16], [17], [18], [19]. Resonance can be partially achieved by geometrical optimization of the structure and fluid volume, i.e., excitation frequency is tuned in relation to the wavelength in a defined volume. To achieve a high efficiency of energy transfer from the electrical power to the effect on the leaching process, a number of development and optimization steps are needed. The most fundamental aspects are:

a) *Methods for the initiation of cavitation bubbles and generate an even distribution of solid mineral particles in the leaching solution.*
b) *Optimization and development of the interconnected resonant systems.*
c) *An energy-efficient ultrasonic excitation principle to create the transient collapse of oscillating cavitation bubbles.*

**Fig. 1:**
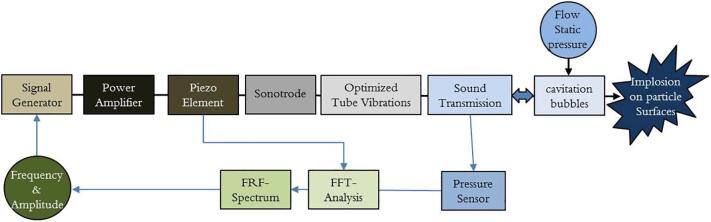
Conversion of electrical power into mechanical energy in the form of vibrations and sound waves to excite and collapse cavitation bubbles on the scheelite particles.

The reactor principle in this investigation is based on a two-step cavitation procedure [14]. First cavitation bubbles are initiated by flow through an orifice and then collapsed by high-intensity ultrasound in a resonant flow through reactor volume. The process can be seen as a chain of components and aspects coupled to each other that needs to be optimized. The goal is to obtain an efficient energy conversion, from electrical power to resonance enhanced ultrasound that creates a mechanical energy impact on the solid particles in the leaching reagent. The input electric power is controlled by a signal generator, and feedback from a pressure measurement in the fluid volume (pressure signal's frequency spectrum). Flow and static pressure are used as control parameters for a stable operating condition as shown in Fig. 1. Minimizing the overall loss factor is necessary for optimal results. To maximize cavitation intensity, a combination of two to three excitation frequencies is probably the best option [11], [12]. Upscaling is possible by extending the reactor tube, and by connecting several tubes in parallel or in series.

## 2 Materials and methods

### 2.1 Material

**Fig. 2:**
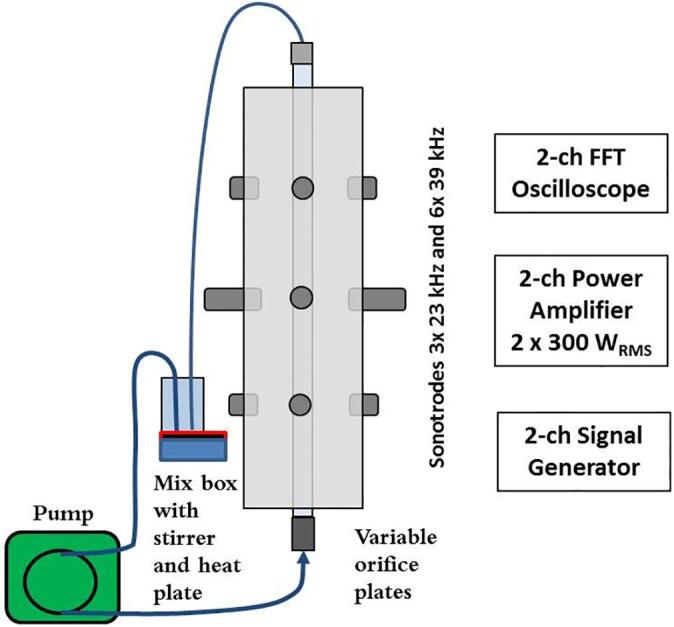
Experimental setup for the extraction of tungsten.

Fig. 2 shows the experimental setup for the extraction of tungsten. The experiments were carried out using a flow through cavitation reactor connected to a 220 ml volume beaker with a magnetic stirrer, a thermometer and a reflux condenser. The beaker was heated using a thermostatic controller in a water bath when the solution flows continuously through the orifice and ultrasound reactor. For each experiment, 11 g scheelite concentrate was added at one time to the agitated NaOH solution (220 ml) at a required temperature. WO_3_ content was measured by ICP − OES. The standard reaction formula is defined as follows:

(1) $$2\text{NaOH}\left(\text{aq}\right)+{\text{CaWO}}_{4}\left(\text{s}\right)={\text{Na}}_{2}{\text{WO}}_{4}\left(\text{aq}\right)+\text{Ca}{\left(\text{OH}\right)}_{2}\left(\text{s}\right)$$

The scheelite concentrate had a measured size distribution of: D10 = 5.87 µm, D50 = 24.96 µm and D90 = 78.43 µm. The mean diameter of particle size was 37.99 µm. The leaching reagent, sodium hydroxide had a concentration of 10 mol/L. The scheelite concentrate was mixed in the leaching reagent at a solid content of 5% (11 g). The total volume for each test was 220 ml. The chemical composition of the scheelite concentrate is given in Table 1.

**Table 1:** Chemical composition of scheelite concentrate.

Elements	WO_3_	Ca	Fe	Mn	Mg	Si	Al
Weight %	75.03	16.4	0.25	0.024	0.0116	0.25	0.07

### 2.2 Reactor design and simulation

**Fig. 3:**
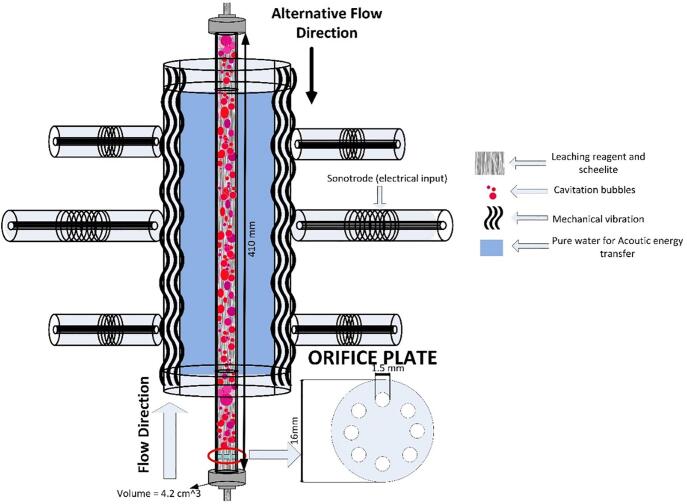
Principal design of the developed hydrodynamic and acoustic cavitation reactor.

Fig. 3 shows the numerically and experimentally optimized reactor concept [14], [20]. The reactor was FE-modeled in 3D using COMSOL Multiphysics®. By extensive numerical optimization, four different resonance phenomena were unified to a coupled resonance [14], [21], [27]. The resonances relate to the sonotrodes; bending wave modes of the reactor wall structure; radial mode of the cylindrical cross-section of the water volume; and the longitudinal standing wave of the water jacket volume.

**Fig. 4:**
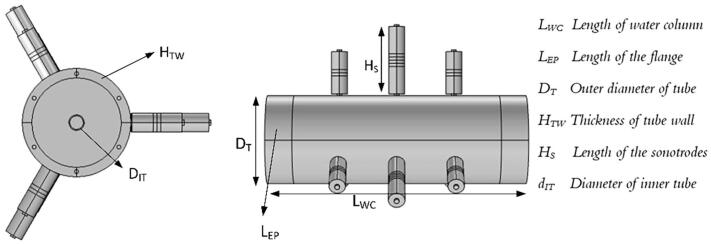
Geometrical configuration of the optimized tube reactor: a) Cross-sectional view and b) Side view, L_WC_ = 342 mm.

The reactor is a rigid shell consisting of a 10 mm stainless steel tube that is excited by nine sonotrodes. The sonotrodes, are resonant structures with integrated piezo-ceramic elements, see Fig. 4. The sonotrodes were excited by dedicated electrical signals around specific frequencies. The outer tube wall vibrations couple to the water jacket at the so-called critical frequency, which relates to material and geometrical properties. At the critical frequency, the bending wave speed equals the speed of sound in the fluid, which gives the most effective coupling between tube vibrations and the sound waves in the fluid. The outer tube diameter was also chosen to get a radial standing wave in the fluid at a frequency above the breathing mode frequency of the tube shell [27].

Powerful excitation of the reactor walls, aim for an efficient and controllable cavitation intensity of the leaching reagent flowing through the inner tube made of PVC (Ø_i_16 mm). The goal of the geometrical design is to create a high vibration amplitude in the outer tube wall to generate a high sound pressure variation within the inner tube of the reactor volume. A powerful cavitation intensity, proportional to the sound pressure variations in the inner tube volume, and hence be achieved energy efficiently. The leaching reagent was modeled by the impedance properties of sodium hydroxide and an experimentally determined loss factor. The system response at resonance is controlled by the loss factors in the system. The loss factor for the resonance mode was determined by impedance measurements represented by the voltage and current signals fed to the sonotrodes using a chirp signal.

**Fig. 5:**
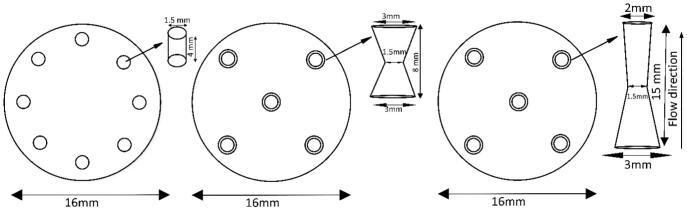
Orifice plates M1, M2 and M3 designed, tested and evaluated during experimental optimization.

To improve energy efficiency, acoustic cavitation was combined with hydrodynamic initiation of cavitation bubbles. Before the mineral suspension flow enters the reactor, cavitation bubbles were initiated by specially designed orifice nozzle plates [23]. The bubble behavior and thereby the generated pressure at the collapse of the cavity for hydrodynamic cavitation depends on the operating conditions and geometry of the mechanical constriction generating cavitation. The effect of operating parameters such as inlet pressure through the system’s orifice, initial cavity size, and the indirect effect of the hole diameter was taken into consideration while designing the three orifice plates shown in Fig. 5 [15], [16].

The geometry was varied by changing the thickness of a 4–15 mm thick cylindrical disc with a number of small holes in three different geometrical patterns as shown in Fig. 5. The basic idea with the orifice plate is to create a local velocity and pressure change, which together with flow friction through the narrow holes, initiates cavitation bubbles [16], [22], [23]. Table 2 shows some of the basic aspects with respect to flow conditions and fluid properties. As indicated by the Reynold numbers, the flow speed through the nozzle is not enough for creating a hydrodynamic cavitation effect. However, by the acoustic excitation of the reactor volume, the pressure fluctuation created, will reduce the cavitation number locally at the orifice and thereby enhancing the cavitation initiation.

**Table 2:** Detailed specifications of NaOH flow through the designed orifice plates.

Plate model	Total orifice area [mm^2^]	Volume flow [lit/min]	Mean flow speed [m/s]	Temp [°C]	Density [kg/m^3^]	Hydraulic diameter [m]	Dynamic Viscosity [mPa s]	Reynolds number
M1	14.1	0.53	0.62	60	1470	0.0015	6.3	206
M2	8.8	0.53	1.00	60	1470	0.0015	6.3	329
M2	8.8	0.85	1.60	80	1470	0.0015	3.2	1040
M3	8.8	0.85	1.60	60	1470	0.0015	6.3	528
M3	8.8	0.85	1.60	80	1470	0.0015	3.2	1040

### 2.3 Experimental methodology

**Fig. 6:**
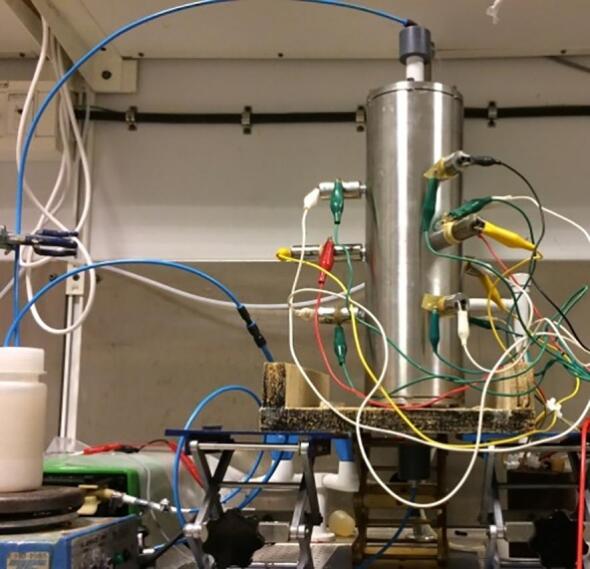
Experimental setup for leaching of scheelite using sodium hydroxide as leaching reagent.

Optimum performance regarding hydrodynamic and acoustic cavitation requires a multi-variate tuning procedure. There is a critical linkage between the leaching solution and solid material regarding cavitation intensity, excitation frequency, and flow conditions. Factors of importance are exposure time, temperature, excitation frequency, input power, static pressure, flow characteristics and concentration. In this analysis, the excitation signals were limited to pure sinusoids at fixed frequencies optimal for the reactor design. All factors were monitored and feedback-controlled during the experiment (Fig. 6). The optimization strategy followed a repeated split plot design (Table 3) focusing on:

• Energy-efficient leaching of tungsten from scheelite concentrate by acoustic cavitation.
• Evaluation of different flow conditions and nozzle geometries regarding hydrodynamic cavitation.

**Table 3:** The repeated split plot design for optimizing leaching by hydrodynamic and acoustic cavitation.

Test (No)	US f_1_ (kHz)	US f_2_ (kHz)	Flow rate(lit/min)	Flow direction	HC(type)	US power (W)(Watt)	Temp (°C)	Exp Time (Hours)
1	23	---	0.53	Bo-Up	No	50	40	5
2a	23	---	0.53	Bo-Up	No	100	60	5
2b	23	---	0.53	Bo-Up	No	200	60	5
3a	23	39	0.53	Bo-Up	No	100 / 100	60	5
3b	23	39	0.53	Bo-Up	No	200 / 200	60	5
4	23	39	0.53	Bo-Up	Yes M1	200 / 200	60	5
5	–	–	0.53	To-Do	No	0	60	5
6	23	39	0,53	To-Do	No	250 / 250	60	5
7	23	39	0,53	To-Do	No	200 / 200	60	5
8	23	39	0,53	To-Do	No	200 / 200	80	5
9	23	39	0,80	To-Do	Yes M2	200 / 200	60	3
10	23	39	0,80	To-Do	Yes M3	200 / 200	60	3
11	23	39	0,80	Bo-Up	Yes M3	200 / 200	80	5

USf_1_: Ultrasound frequency one; USf_2_: Ultrasound frequency second; Bo-Up: flow bottom to top; To-Do: flow top to bottom; HC: Hydrodynamic cavitation with orifice plates; EXP Time: time of exposure.

For each test, samples from the leaching reagent were collected every hour. The flow circuit including the reactor system had a total volume of 220 ml, where 33% were activated by acoustic cavitation. Each sample taken was 1.5 ml, using a pipette inserted into the temperature controlled mixing container (120 ml). The collected sample was then filtered using a 0.45 µm syringe-filter setup. The filtered solution was analyzed for tungsten through ICP-OES (Thermo Fisher®).

## 3 Results

The ultrasound cavitation reactor was developed to handle a highly concentrated leaching reagent (10 M sodium hydroxide). Tests were conducted at 38, 60 and 80 °C at varying flow conditions, input powers and excitation frequencies. The excitation signals were adapted to the reactor's experimentally optimized frequency response at around 23 kHz and 40 kHz. The temperatures of 60° and 80 °C were selected for comparison with chemical leaching reference data.

**Fig. 7:**
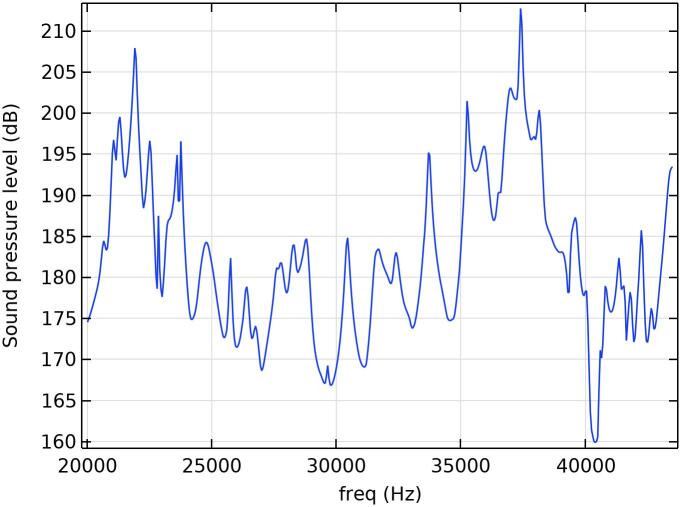
Estimated linear frequency response for FE-modeled and optimized cavitation reactor. The pressure response is determined as an average within the volume where the leaching reagent flows through, with a loss factor of 2% (experimentally determined).

Fig. 7 shows the simulated frequency response of the reactor when excited with two different types of sonotrodes tuned to 22 kHz and 37 kHz. The calculations were performed with stepwise sinusoidal excitation, giving the linear system response at each frequency. However, in reality the rms-value at the frequency of pure sinusoidal excitation reaches about 215 dB (63 kPa). Due to non-linear effects, the total frequency response of pure tone excitation is seen as a wide harmonic spectrum well beyond 100 kHz, where the harmonic amplitude values reach levels of 212–218 dB. The pressure signal, measured in the center of the water filled reactor, low pass filtered below 100 kHz, reaches positive peak values of 400 kPa at 200 W input power.

**Fig. 8:**
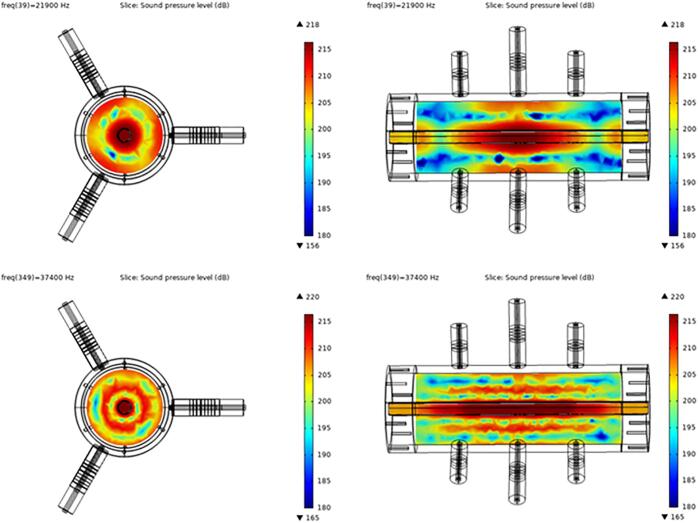
FE-calculated pressure response of optimized reactor geometry at a) 21.9 kHz and b) 37.4 kHz. The sound pressure level relates to linear acoustic modeling at low input power.

At the resonance frequencies, the corresponding modes generates high cavitation intensity in the central region of the reactor as simulated in Fig. 8. In the experiments, maximum input power at the excitation frequencies was 250 W at 22.6 kHz, and 200 W at around 40 kHz. The power conversion efficiency of the water-filled reactor and no flow, was 36% (determined by calorimetric test [21], [24], [26]). The performance was also verified by foil tests.

Most of sodium tungstate (Na_2_WO_4_) is produced in solid form because of its low solubility. The solubility of Na_2_WO_4_ reduces and therefore more Na_2_WO_4_ crystallizes with a rise in NaOH concentration that activates reaction Eq. (1) in to the right state, Eq. (2) [4].

(2) $$2{NaOH}_{\left(aq\right)}+{{CaWO}_{4}}_{\left(s\right)}Na_{2}WO_{4\left(s\right)}={Na}_{2}{{WO}_{4}}_{\left(aq\right)}+Ca{\left(OH\right)}_{2\left(s\right)}$$

The results summarized in Table 4, show that the recovery rate is dependent on process temperature, input electrical power (proportional to ultrasound intensity) and flow conditions. The results from 5 h exposure span from 4.6% recovery of WO_3_ in case of chemical reactor leaching without ultrasound at 38 °C, to 67.1% in case of hydrodynamic and acoustic cavitation at 80 °C. In the latter case (Test 11), applying 370 W input electrical power at two different ultrasound frequencies and hydrodynamic cavitation, the results obtained are in line with Zhao et al. [28].

**Table 4:** Experimental details and summary of results with respect to tungsten (WO_3_) recovery [%].

Test (No)	US f_1_ (kHz)	US f_2_ (kHz)	Flow(l/min)	Flow Dir	HC	ElectricPower (W)	Temp (°C)	Exp Time (h)	CHEM^d^ WO_3_(%)	HAC^e^ WO_3_ (%)
1	22.9	---	0.53	BoUp	No	36.2	38	5	4.6	6.4
2a	23.0	---	0.53	BoUp	No	104	60	5	24.1	23.1
2b	23.4	---	0.53	BoUp	No	210	60	5	24.1	25.5
3a	23.1	39.0	0.53	BoUp	No	120/110	55–60	5	24.1	21.4
3b	24.1	43.1	0.53	BoUp	No	220 /200	60	5	24.1	29.3
4	23.8	43.5	0.53	BoUp	YesM1	190 /170	60	5	24.1	25.7
5^a^	–	–	0.53	ToDo	No	0	52–60	5	24.1	12.1
6^a^^,^^b^	24.1	41.5	0.54	ToDo	No	250/220	60	5	24.1	19.9
7^a^^,^^b^	22.3	38.5	0.54	ToDo	No	200/200	60	5	24.1	21.0
8^a^^,^^b^	22.1	39.2	0.54	ToDo	No	200/200	77	3	18.7	32.2
9^a^^,^^b^	22.2	38.5	0.80	ToDo	YesM2	200/200	60–65	3	18.7	24.0
10^a^^,^^b^	22.1	39.2	0.80	ToDo	YesM3	200/200	60–65	3	18.7	26.9
11^c^	22.3	39.6	0.80	BoUp	YesM3	220/150	75–80	5	32.9	67.1

a Experiments with different flow direction (top/down).

b Re-designed sonotrodes, since piezo properties changed during long term exposure.

c Tests using bottom-up flow direction, where bubbles go with the flow and heavier particles stay for a longer time.

d CHEM: Chemical leaching recovery of tungsten by stirring in a beaker at various temperatures in ambient conditions.

e HAC: Leaching recovery with hydrodynamic and acoustic cavitation.

**Fig. 9:**
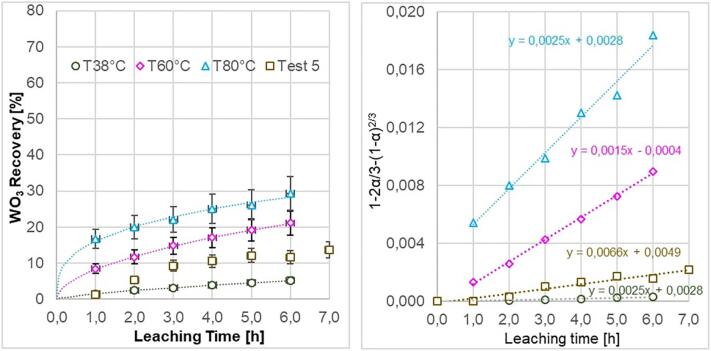
a) Leaching recovery of tungsten (WO_3_) from scheelite concentrate in the absence of hydrodynamic and acoustic cavitation. b) Linearized recovery rate according to a shrinking core model where the largest resistance to the process is the diffusion through the boundary layer.

Fig. 9a shows the temperature effect on the recovery rate in case of chemical leaching in a beaker with magnetic stirrer and in absence of ultrasound. The recovery rate for 5 h increased from 3.7% to 32.9% at 38 °C to 80 °C respectively. Error bars reflect the 95% confidence interval of the ICP-OES analysis. The data is well fitted (r^2^ > 0.95) to a regression model of the type y = ax^b^ shown as dotted lines. Fig. 9b represents the linearized recovery rate according to a shrinking core model where the largest resistance to the process is the diffusion through the boundary layer [28]. This shrinking core model, y = 1-2α/3-(1-α)^2/3^ is only valid when there is a homogeneity in the particle sizes. Even though the particle sizes used in this analysis are heterogeneous, the shrinking core model helps to interpret the recovery rates. This can be observed in the case of chemical leaching shown in Fig. 9b. However at 80 °C, the wide spread of particle sizes in the concentrate solution and the absence of diffusive boundary layer on the particles gives a greater recovery rate during the first hour.

In case of Test 5, when the reagent solution flows through the reactor in the absence of ultrasound at (52–60 °C), the recovery of tungsten (WO_3_) is reduced in comparison to chemical leaching. One reason for reduced recovery was due to difficulties in maintaining an ambient temperature of 60 °C when the reagent passes through the unexcited reactor volume. In the first two hours, temperature varied and dropped down to 52 °C but became more stable over time. The other reason was due to the lack of mixing in the flow circuit since 100 ml of leaching reagent was outside the beaker with magnetic stirrer.

**Fig. 10:**
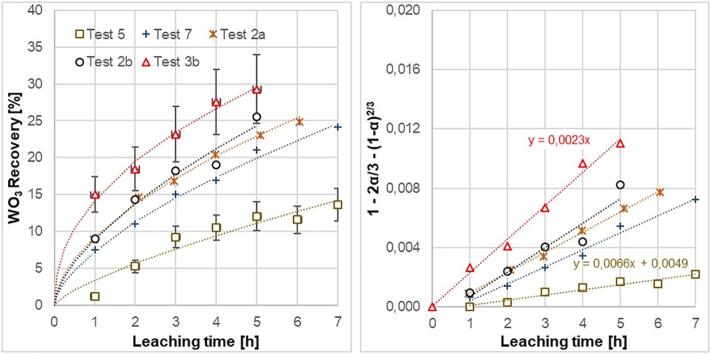
a) Leaching recovery of tungsten (WO_3_) from scheelite concentrate in the presence of acoustic cavitation at 60 °C. b) Recovery data linearized according to a shrinking core model, where the largest resistance to the process is the diffusion through the boundary layer.

Fig. 10 shows the acoustic cavitation effect of the flow through reactor at 60 °C. At 100 W power and a single frequency excitation (23.4 kHz), the recovery rate after 5 h was 23.1% (Test 2a). Increasing input power to 210 W at a single frequency (23.1 kHz) increased the recovery rate to 25.5% (Test 2b). A doubling of the input power to 420 W, by excitation of two ultrasound frequencies, increases the recovery rate to 29.3% (Test 3b). In Test 3a excitation frequencies were not optimal, and therefore shifted upwards in Test 3b. Especially the second and higher frequency gave a better pressure response and cavitation efficiency. Under similar acoustic cavitation conditions, the cavitation effect is better in the case of bottom-up flow (Test 2b) compared to top down flow direction (Test 7). Test 3b is significantly better than chemical leaching, which indicates an efficient cavitation effect in the reactor volume. The cavitation effect is even greater than indicated since the ultrasound reactor volume only represents 33% of the total flow circuit volume.

**Fig. 11:**
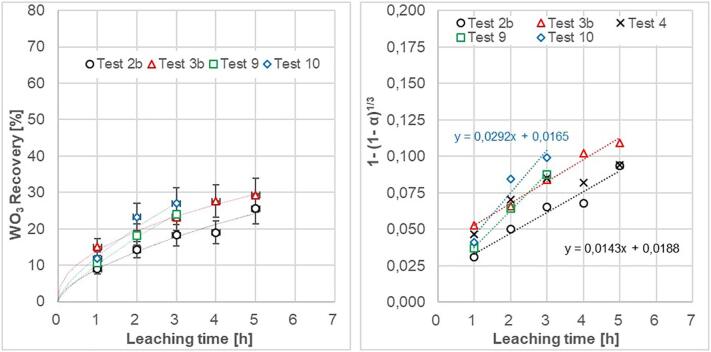
a) Leaching recovery of tungsten from scheelite at 60 °C with hydrodynamic and acoustic cavitation. b) Recovery data transformed with respect to a shrinking core model controlled by the surface chemical reaction rate. The results of Test 4 are included to demonstrate the problem of bigger particles being trapped in front of nozzle M1.

Fig. 11 shows the effect of hydrodynamic and acoustic cavitation intensity on the recovery rate of tungsten. Results of Test 9 and 10 shows the improved leaching effect by introducing hydrodynamic cavitation. Fig. 11b represents the linearization of the leaching data describing the kinetics of a shrinking core model [28], [29]. The recovery rate of WO_3_ has been transformed by y = 1-(1-WO_3_%/100)^1/3^. The slope of the linearized function represents the reaction rate constant for a specific experimental condition. The hydrodynamic effect generates a greater slope, which corresponds to better initiation of cavitation bubbles, which leads to improved mixing, reduction of diffusion layers and increased number of collisions.

In the case of hydrodynamic cavitation, Fig. 11b does not fully explore the benefit of the bottom-up flow direction. After 3 h, with the top-down flow, when using orifice plates M2 and M3, gave recovery rate 24,0% and 26.9% respectively compared to bottom-up flow with orifice plate M1 of 23.3%. The reason to believe in the bottom-up flow direction was due to the fact that nozzle M1 did not work properly. When the reactor was dismantled after Test 4, it was seen that bigger particles (volume < 0.5 ml) had stacked in the volume at the inlet of orifice plate. Therefore, the orifice plate was replaced by M2 and M3 (Fig. 5). In similar test conditions, M3 gave better recovery than M2 at 60 °C. Test 11 was therefore conducted with bottom-up flow direction using orifice plate M3 at a temperature of 80 °C.

**Fig. 12:**
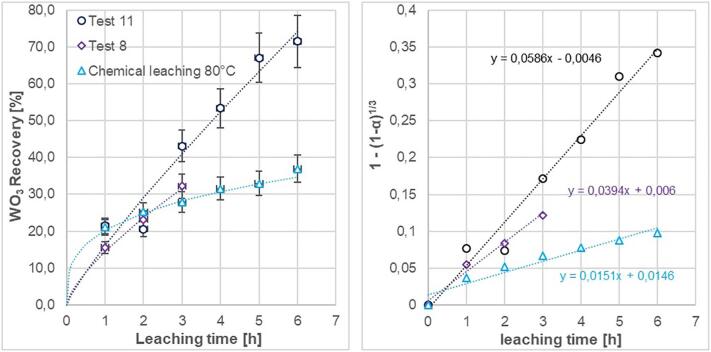
a) Leaching recovery of tungsten (WO_3_) from scheelite at 80 °C, with hydrodynamic and acoustic cavitation compared to chemical leaching. b) Reaction rates for the different conditions. Recovery data are transformed with respect to a shrinking core model controlled by the surface chemical reaction rate.

Fig. 12, shows the recovery rate at 80 *°*C comparing two different hydrodynamic and acoustic cavitation-leaching conditions with chemical leaching. Test 11, using the orifice plate M3 and having a flow against gravity, gives a recovery rate that is two times better than chemical leaching without ultrasound. The result of Test 11 confirms the choices made and showed a significant improvement in the recovery rate of WO_3_ (67.1% after 5 h and 71.5% after 6 h). Due to the geometrical differences M3 generates higher turbulence intensity, which thereby leads to a better mixing rate than M2 [16], [25]. The high intensity of turbulence is obtained as the fluid flows through the orifice in to the contraction zone of the orifice, which generates a sudden increase in velocity head at the expense of pressure head. During re-expansion or retardation of the fluid, the flow is separated at the lower end of the orifice and hence the eddies are generated. This flow phenomenon becomes more pronounced in case of orifice plate M3. The motion of the eddies causes an increase in turbulence intensity, leading to frictional losses which in combination with pressure fluctuations generated by the ultrasound give higher efficiency of leaching recovery [16], [25].

The low recovery ratio for Test 11 at 2 h is most likely caused by a temporary sedimentation or coagulation effect creating an uneven distribution of the leaching reagents in the flow circuit. By that, a greater proportion of the scheelite concentrate got temporally stacked in the flow circuit and did not enter the reactor nor the beaker where samples were taken.

The 71.5% tungsten (WO_3_) recovery was obtained by an energy supplement equivalent to 130 kWh/kg scheelite concentrate. The chemical leaching test conducted, when using a magnetic stirrer at 600 rpm in the beaker of 220 ml used at the same temperature and exposure time, gave a recovery rate of 36,9%. In case of autoclave leaching at very high temperature and pressure greater recovery rates are achieved [4]. However, results from Test 11 shows that the combination of a mild hydrodynamic effect and acoustic cavitation has a great potential for sustainable leaching of tungsten. The results of Test 11, conducted in an open flow circuit are as good as the recovery rate of autoclave leaching of scheelite in laboratory scale [29].

## 4 Discussion

A limitation in the experimental setup was that the volume of the cavitation reactor only represented 33% of the total circulating volume. This means that the energy supplied to the material to be leached can be more than doubled in an up-scaled reactor geometry (longer tube), thereby increasing the yield for a given exposure time. A longer reactor (vertical orientation) also means a natural increase in the static pressure in the system.

A specific problem during tests was overheating. One aspect was a too soft piezo material (pzt27) that became stiffer over time, which altered the resonance frequencies of the sonotrodes. Therefore, the sonotrodes needed to be re-tuned after Test 4 by increasing the length of the supporting masses by 2.0 mm. The other aspect of overheating was due to too high input power and the fact that the process temperature of 80 °C requires additional cooling of the sonotrodes.

A possible improvement when hydrodynamic cavitation becomes more pronounced is to tune the higher excitation frequency differently. Despite the good recovery rate of Test 11, the input power at 39 kHz was low, due to some impedance miss-match. One hypothesis is that the impedance of the inner tube structure changed, which had a negative impact on the response of the sonotrodes used for the excitation frequency 39 kHz.

In general, an even higher process temperature may be needed, which require better heat insulation and over pressure of the water jacket inside the reactor. However, the leaching reagent may not need over pressure, if a process temperature of 80–90°is enough. The optimal process temperature is in proportion to the boiling temperature of the leaching reagent (137 °C for NaOH, 10 M). Finally, a higher flow rate is may be needed to take full advantage of the hydrodynamic cavitation effect induced by the designed orifice plate M3.

With respect to hydrodynamic cavitation, the tests showed that hydrodynamic cavitation alone did not produce any significant improvement in the leaching recovery rate. It may be obvious since the limited flow rate was unable to reach a high enough Reynolds number (Re = 1040 in Test 11). However, when acoustic cavitation is used, the pressure fluctuations in the reactor give varying pressure at the orifice plate, which reduces the cavitation number below one, which enables the initiation of cavitation bubbles.

## 5 Conclusions

The objective of the ongoing project was to fine-tune a scalable reactor concept with respect to leaching of scheelite, a mineral known to be hard to leach. The leaching reagent was a mix of sodium hydroxide (NaOH, 10 M) and 5% (11 g) scheelite concentrate. The focus was on optimizing the recovery of tungsten on the basis of acoustic cavitation intensity, process temperature and flow conditions.

The best test result shows that an energy supplement by hydrodynamic and acoustic cavitation of 131 kWh/kg concentrate, gives a leaching recovery by 71.5% after 6 h exposure at 80 °C. The result can be compared to a 36.7% recovery when using conventional stirring at the same exposure time and temperature. The supplemented energy by acoustic cavitation seems to increase the recovery rate proportional to higher process temperatures. This is most likely caused by collapsing bubbles, which generates an increase of microscale temperatures, number of collisions and removing diffusion layers by shockwaves and micro-jets.

However, longer exposure time and a higher process temperature are necessary to achieve a leaching recovery rate corresponding to today's autoclave technology. The long-term target is to maximize the recovery rate of tungsten (>90%) from the scheelite concentrate. By minor modifications, the reactor principle can be used for various applications.

## CRediT authorship contribution statement

**Örjan Johansson:** Conceptualization, Methodology, Writing - original draft, Formal analysis, Funding acquisition, Visualization, Validation, Supervision, Project administration. **Taraka Pamidi:** Software, Methodology, Formal analysis, Investigation, Data curation, Visualization, Writing - review & editing. **Vijay Shankar:** Supervision, Formal analysis, Writing - review & editing.

## Declaration of Competing Interest

The authors declare that they have no known competing financial interests or personal relationships that could have appeared to influence the work reported in this paper.
